# Resistance Switching Behavior in Rectangle-Nano-Pattern SrTiO_3_ Induced by Simple Annealing

**DOI:** 10.3390/ma12223698

**Published:** 2019-11-09

**Authors:** Xiaxia Liao, Yufeng Zhang, Jiaou Wang, Junyong Kang, Jinbin Zhang, Jizheng Wang, Jincheng Zheng, Huiqiong Wang

**Affiliations:** 1 School of Materials Science and Engineering, Nanchang University, Nanchang 330031, China; liaoxiaxia@ncu.edu.cn; 2 Department of Physics, and Collaborative Innovation Center for Optoelectronic Semiconductors and Efficient Devices, Xiamen University, Xiamen 361005, China; yufengzhang@xmu.edu.cn (Y.Z.); jykang@xmu.edu.cn (J.K.); 3 Beijing National Laboratory for Molecular Sciences, Key Laboratory of Organic Solids, Institute of Chemistry, Chinese Academy of Sciences, Beijing 100190, China; jizheng@iccas.ac.cn; 4 Beijing Synchrotron Radiation Facility, Institute of High Energy Physics, Chinese Academy of Science, Beijing 100049, China; wangjo@ihep.ac.cn; 5 College of Materials, Xiamen University, Xiamen 361005, China; jbzhang@xmu.edu.cn; 6 Institute of Artificial Intelligence, Xiamen University Malaysia, Sepang 43900, Malaysia

**Keywords:** resistance switch, strontium titanium oxide, oxygen vacancy, annealing, rectangle-nano-pattern

## Abstract

The tunability of semi-conductivity in SrTiO_3_ single crystal substrates has been realized by a simple encapsulated annealing method under argon atmosphere. This high temperature annealing-induced property changes are characterized by the transmission spectra, scanning electron microscopy (SEM) and synchrotron-based X-ray absorption (XAS). We find the optical property is strongly influenced by the annealing time (with significant decrease of transmittance). A sub gap absorption at ~427 nm is detected which is attributed to the introduction of oxygen vacancy. Interestingly, in the SEM images, annealing-induced regularly rectangle nano-patterns are directly observed which is contributed to the conducting filaments. The XAS of O K-edge spectra shows the changes of electronic structure by annealing. Very importantly, resistance switching response is displayed in the annealed SrTiO_3_ single crystal. This suggests a possible simplified route to tune the conductivity of SrTiO_3_ and further develop novel resistance switching materials.

## 1. Introduction

Recently, resistive random access memories (RRAMs), possessing excellent miniaturization potential, fast operation speed and strong endurance, have attracted considerable attentions in virtue of the most promising alternative to the high-density and low-cost future data storage in the non-volatile memory market and logic circuits [1,2]. RRAMs are based on the resistive switching (RS) effect, where the tunable resistance of materials can be reversibly controlled under an electric field. However, the switching mechanism is still under debate. In macroscopic respect, the Schottky barrier contact between metals and oxide semiconductors is an important research branch, which is thought to be critical to tune the resistance state [3,4,5]. In the microscopic respect, dislocation-related conductive filaments are confirmed to be the main contributions [6,7,8,9,10,11] while Celano et al. observed the non-filamentary resistive switching (also called areal switching) behavior [12]. Therefore, how to deliberately create the resistive switching property is a fundamental issue in obtaining a stable resistive device.

The perovskite oxides become the intensely investigated materials due to the easy manipulation of conductivity [13]. Neighbouring atom substitution is the regular method to induce carrier doping to change the insulating natural property, such as Nb:SrTiO_3_, La_0.7_Sr_0.3_MnO_3_, Fe:SrTiO_3_ etc., systems [1,3,14]. Additionally, the post treatments, like high temperature annealing, laser irradiation, and ion bombardment are the common ways to introduce vacancies/dislocations in the materials [8,15,16,17,18]. Pan et al. observed resistive switching behavior in SrTiO_3_ single crystal after a relative high flux of laser irradiation. The generation of enough oxygen vacancies forms the conductive filaments [18]. Psiuk et al. reported that the conductive layer formed effectively through surface modification under low energy Ar^+^ sputtering [16]. To reveal the interaction between oxygen vacancies and dislocations in SrTiO_3_ single crystals, Schraknepper et al. carried out the oxygen tracer diffusion experiment above 1000 K. They proposed three features in the SrTiO_3_ layer depending on the depth from surface to bulk and the discovery arrays of dislocations [7]. Crespillo et al. applied another useful technique, the MeV ion irradiation to create the oxygen vacancy disorder in the SrTiO_3_ and reveal the modification of the electronic properties [19,20,21]. The optical identification of oxygen vacancies in the SrTiO_3_ has been clearly demonstrated through a red luminescence signature at 2.0 eV, which is related to the self-trapped Ti^3+^ polarons at isolated oxygen vacancies [19,20,21,22]. A 2.8 eV emission band has been detected and attributed to the recombination of free (conduction) electrons with an in-gap level, identified as the ground state level of the self-trapped excition center [23].

Compared to the conventional thermal annealing in vacuum, which is energy-consuming and requires a complicated setup to obtain a high vacuum level, we employ an easy and fast operation for annealing. A simple oxyacetylene heating system is used to seal the sample into a quartz capsule first, then the samples are annealed in the ambient atmosphere. The details are described in the experimental section. Our results show that this simple, energy-conservation method not only generates oxygen vacancies, but also creates interesting rectangle-shape nano-patterns which introduce the resistance switching behavior in the SrTiO_3_ single crystals.

## 2. Materials and Methods 

One-side polished SrTiO_3_ (001) single crystal substrates (10 × 5 × 0.5 mm^3^) are purchased from Hefei Kejing Materials Technology Co., LTD (Hefei, China). All the SrTiO_3_ substrates are cleaned in alcohol by ultrasonic and dried; afterwards they are put into every quartz capsule and evacuated twice, then backfilled with argon gas and sealed with the pressure around 10^4^ Pa. The quartz capsules with the samples in are placed into the furnace and annealed at 1000 °C for 6 h, 12 h, 18 h, and 24 h, respectively. Therefore, the four annealed SrTiO_3_ substrates are labeled as “6 h”, “12 h”, “18 h”, and “24 h” hereinafter. An “as received” SrTiO_3_ substrate (without annealing) is also measured for comparison. The morphologies of the formed surface structures are characterized by SEM (LEO 1530, Zeiss, Jena, Germany), with an operating voltage of 20 kV) and AFM (carried out in air by using the Multimode Nanoscope III equipment in tapping mode). XAS spectra are acquired in the 4B9B beamline in Beijing Synchrotron Radiation Facility (BSRF). The 4B9B beamline is equipped with Scienta R4000 electron energy analyzer (VG Scienta, Uppsala, Sweden) and is operated in ultra-high vacuum conditions: base pressure in the analysis chamber is ~1.0 × 10^−10^ Torr. O K-edge are collected from 525 eV to 560 eV via the total electron yield mode with the energy step of 0.2 eV. The XAS spectra shown here are smoothed but with the original shape. For the fabrication of the RRAM device, the patterned Au electrode with the size of 5 mm × 1 mm is thermally deposited and the Ag electrode is silver glue with the diameter of about 2 mm. The current voltage curves are measured by a Keithley 2400 source meter.

## 3. Results and Discussions

**Figure 1:**
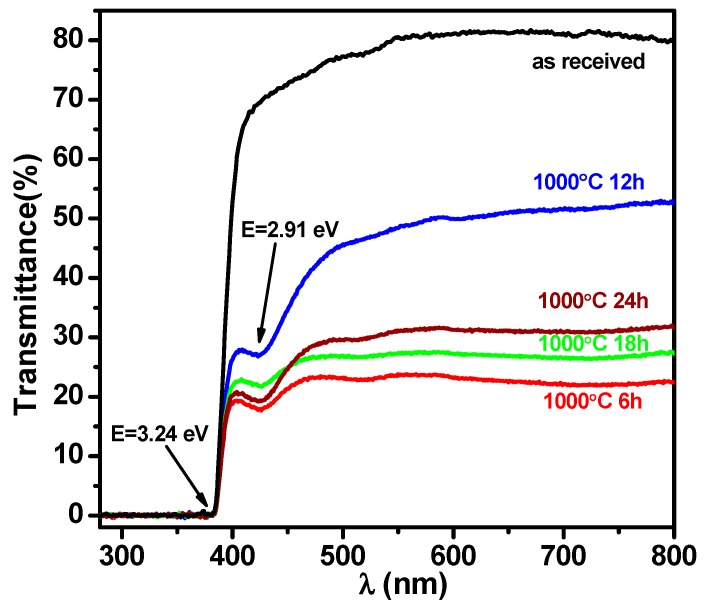
The transmittance of SrTiO_3_ single crystals after 6, 12, 18, 24 h annealing compared to the “as received” one.

High temperature annealing imposes a strong influence on the SrTiO_3_ single crystal. As we know, the stoichiometric SrTiO_3_ is a white transparent insulator with a bandgap of 3.2 eV [24]. After high temperature annealing, the color of four SrTiO_3_ samples changes from white to yellowish or blackish which indicates the introduction of defects and the enhancement of absorption coefficient as the annealing induced reduction. As shown in Figure 1, the optical transmittance of the “as received” SrTiO_3_ substrate reaches 80% comparing to a significant decrease of the annealed ones. For the “6 h” sample, the optical transmittance drops to as low as 20%, while for the “12 h”, “18 h” and the “24 h” samples, the optical transmittance increases back to around 50% and 30% under the extensive annealing. Therefore, the optical transmittances are not inversely proportional to the annealing time. As discussed in the previous reports, high temperature annealing in the reduced atmosphere is the common method to introduce the oxygen vacancies as well as the conductivity of the oxides. In our work, the annealing occurs in the low pressure of argon atmosphere which plays the similar function. However, the oxygen atoms will not escape from the oxide surface with prolonged annealing time. Szot et al. observed this phenomena in SrTiO_3_ single crystal under vacuum annealing and they revealed that it was a self-healing phenomenon in which the initially generated density of vacancy defects and related charge carriers are dramatically decreased at 800 °C [25]. Hence, we speculate that self-healing behavior occurs during our annealing procedure which creates different concentrations of oxygen vacancies. Additionally, the varied optical transmittances are the evidence of the concentration of the defects. In Figure 1, the derived bandgap is 3.24 eV which suggests a stoichiometric SrTiO_3_ single crystal. Furthermore, a sub-absorption located at 2.91 eV (marked by the black arrow) is created under high temperature annealing. This sub band is 0.3 eV narrower than the band gap of stoichiometric SrTiO_3_ which indicates the existence of defect states in the bandgap. Cuong et al. obtained the similar results by using the first principle calculations in oxygen-deficiency SrTiO_3_ [26].

**Figure 2:**
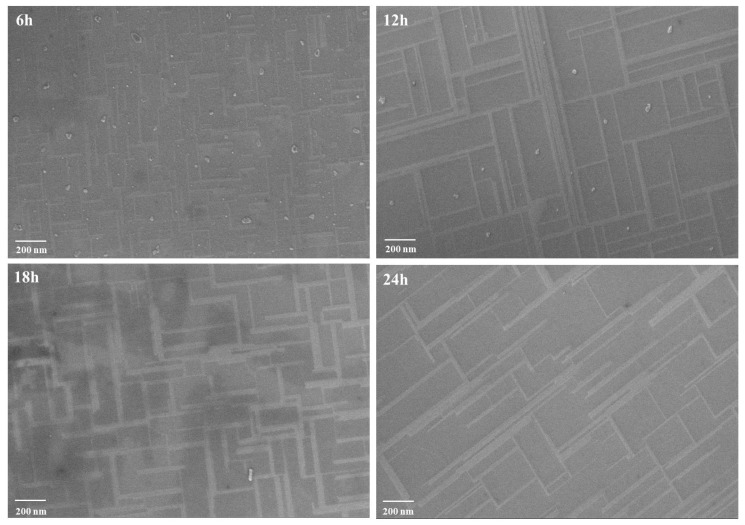
The SEM images of SrTiO_3_ single crystals after 6, 12, 18, 24 h annealing.

In order to fully understand the influence of high temperature annealing on the surface morphology, SEM images are taken (shown in Figure 2). For comparison, the “as received” SrTiO_3_ sample is measured (not shown here). The surface is smooth without obvious microstructure which is the initial appearance of the commercialized polished substrate. After annealing, regular rectangle-shape nanostructure patterns are clearly observed. For the “6 h” and “18 h” samples, short and crossed bright stripes are created; while for the “12 h” and “24 h” samples, the stripes become sharp and elongated. This feature has been reported in previous publications. Szot et al. directly observed a network of extended defects/dislocations distributed for the first tens of μm from the surface to bulk of SrTiO_3_ single crystal by local-conductivity atomic force microscopy (LC-AFM) [10,25]. Shablaev et al. studied the unsteady-state in the high resistance SrTiO_3_ because of the network of conductive nanowires [27]. In the uniaxial compression experiments, a general increase of dislocation densities appears when SrTiO_3_ single crystal is deformed at higher temperature [17]. Dislocations are always introduced by high temperature annealing as well as the distorted lattice in SrTiO_3_ [25]. Combined with our work on the SrTiO_3-δ_ system, we find that oxygen vacancies tend to align into a chain by using first-principles calculations and the creation of lattice distortion [28]. Therefore, we speculate that these nanostructured-crossed patterns on the SrTiO_3_ are correlated to the defect dislocations. As exhibited in Figure 1, the “6 h” and “18 h” samples display low transmittance. The condensed and short network patterns suggest more defects are induced which well explains this observation.

**Figure 3:**
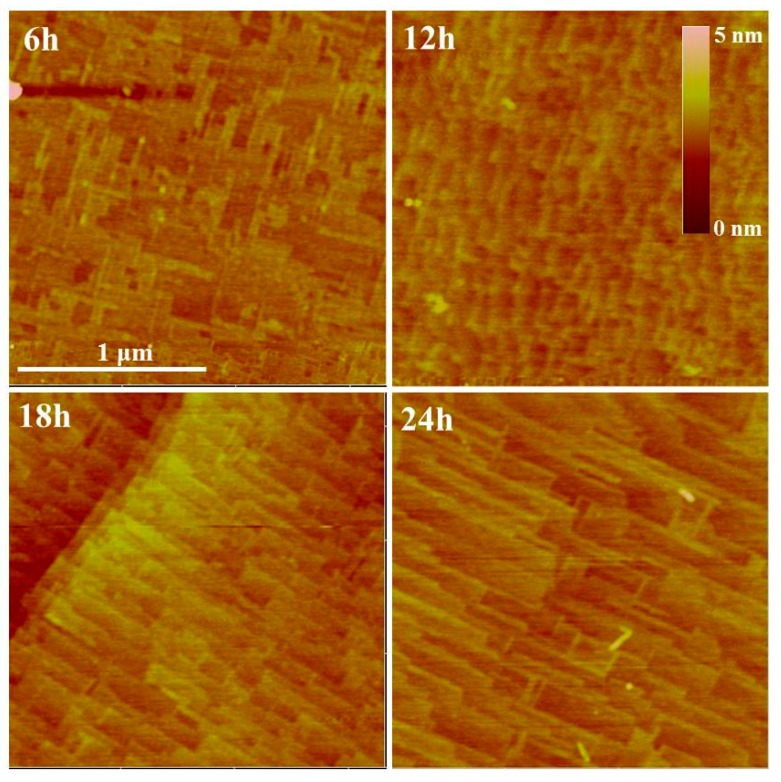
The AFM images of SrTiO_3_ single crystals after 6, 12, 18, 24 h annealing.

Furthermore, AFM measurement (shown in Figure 3) is conducted to investigate the surface roughness. High temperature annealing imposes a little effect on the roughness. The surface is still quite flat with the RMS roughness of the four SrTiO_3_ crystals as 0.28 nm, 0.20 nm, 0.53 nm and 0.19 nm, respectively, which is quite different from obvious surface roughness generation by laser annealing [18]. Small surface roughness facilitates the electrode contact. The sort of large roughness of the “18 h” might be due to the scratch on the surface. The stripes-like patterns can also be seen.

**Figure 4:**
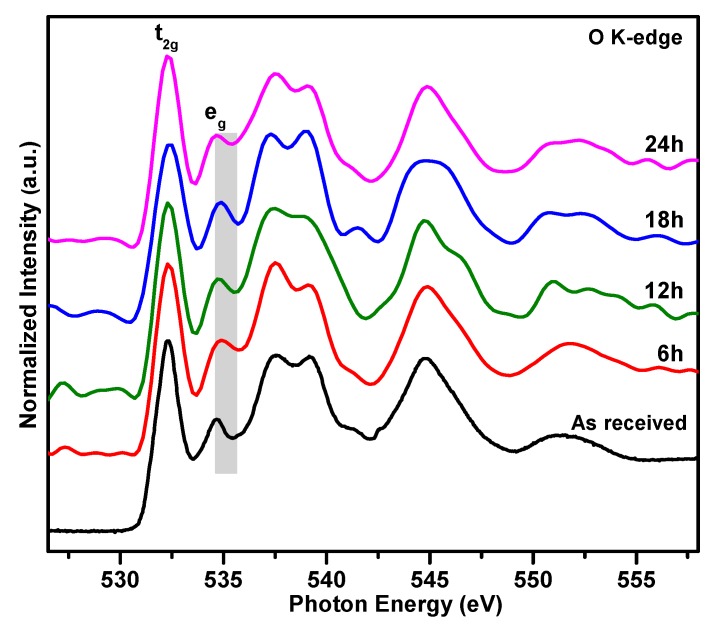
XAS spectra of O K-edge features of SrTiO_3_ single crystals after 6, 12, 18, 24 h annealing.

To further exam how the high temperature annealing impacts the electronic structure of SrTiO_3_ crystals, synchrotron-based XAS of O K-edge is carried out as shown in Figure 4. O K-edge represents the transition from O 1s core level to the unoccupied O 2p level. Due to the hybridization effect, this absorption spectrum is divided into three regions [29,30,31]. The region above the threshold is assigned to the transition from O 1s states to O 2p states hybridized with Ti 3d states. The energy between 536–542 eV is attributed to the transition from O 1s orbitals to hybridized O 2p + Sr 4d orbitals. The broadening spectra at the higher energy region are predominated by the excitation to Ti 4sp states. Due to the crystal field effect, the split of Ti 3d two bands (t_2g_ and e_g_) is about 2.4 eV, which is in good agreement with the report on SrTiO_3_ films [30]. As highlighted by the grey rectangle background in Figure 3, the e_g_ peak is broadening at the high energy side after annealing compared to the “as received” SrTiO_3_. Gautam el al. suggested that the e_g_ band is quite sensitive to the local environment, since in the octahedron the central Ti e_g_ orbitals point directly toward to the 2p orbitals of the surrounding O atoms [29]. Palina et al. reported that the generation of defects such as oxygen vacancies, induces delocalized electrons which leads to the broadening of the corresponding resonance peak [32]. Therefore, our O K-edge further reflects that the high temperature annealing introduces oxygen defects.

**Figure 5:**
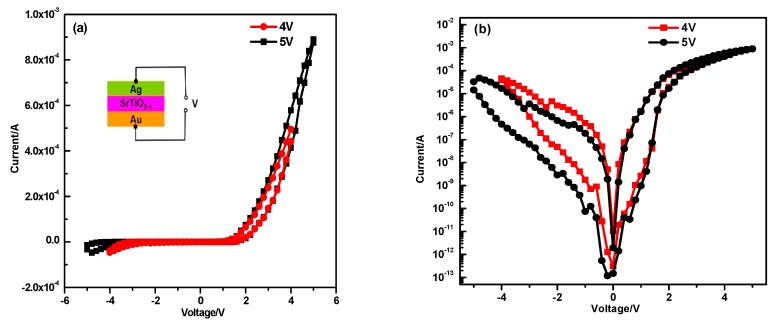
(**a**) The I-V characteristic and (**b**) semi-log I-V curve of the “24 h” sample in the configuration of Au/SrTiO_3_/Ag.

From the above discussion, high temperature annealing creates defects in the SrTiO_3_ which should significantly enhance the corresponding conductivity. To gain further insight into the annealing effect, a “24h” SrTiO_3_-based device (inset of Figure 5a) is fabricated to measure its resistance switch behavior. Resistive switching behavior are measured upon applied voltages. The sweep mode follows 0→5 V→0→−5 V→0 cycle with the bias voltage of 4 V and 5 V. As shown in Figure 5a, an asymmetric rectifying I-V characteristics with large hysteresis is observed. In semi log scale, a butterfly-like hysteresis loop is exhibited in Figure 5b. This I-V behavior demonstrates that the resistance switch is induced by the high temperature and converts between the high resistance state and the low resistance state. The current increases with the bias voltage of 5 V to 4 V. Bailey et al. initiated the highly insulating SrTiO_3_ film to a semiconducting film by a bias-applied (2–5 V) sweep at the first initial stage like electron-forming in many reports [18,33,34]. They also observed that the device current and hysteresis behavior were strongly dependent on the bias-voltage. The asymmetric I-V feature might be explained by the different interface barrier height due to the application of electrodes (Au and Ag) [35,36]. The other SrTiO_3_ samples with different annealing times also displayed similar resistive switching behavior (not shown here). Due to the fast release (within 5 min) of oxygen vacancies from SrTiO_3_ single crystal [25], more deliberately annealing procedures are needed to further analyze the effect of annealing time for resistive switching.

For the SrTiO_3_-based resistance switch, the basic step is the introduction of defects to increase the conductivity by various treatments, while the mechanism is still without consensus [33,37,38,39,40]. The generation of dislocation/vacancies is confirmed in the samples and reached a well agreement. However, the unclear part is how these defect movements/transportations contribute to the resistance switch. Due to the donor character of the oxygen vacancy, Pan et al. reported the newly formed networks conductive filaments and the oxygen vacancies contribute to the electron carriers [18]. This viewpoint can be further supported by the lower oxygen vacancy formation energy at the dislocation core [9]. Therefore, the local changes of electrical properties induced by the dislocations was not because of the preferably faster kinetics of oxygen migration (pipe diffusion), but simply because of the structure-driven easier reducibility [9]. Rodenbucher et al. observed that the current is channeled along the extend dislocations directly by thermal microscopy. From our results, we prefer to attribute the resistance switch to the oxygen vacancies formed filaments based on the direct observation of the regular rectangle nanostructures in the SEM images.

## 4. Conclusions

In our work, we have obtained the reduced SrTiO_3_ single crystal by applying a simple and feasible annealing method. The small absorption above the absorption edge indicates the creation of defect states in the bandgap. Combing the regular rectangle nanostructures in the SEM image and electronic structure changes in the X-ray absorption spectra of O K-edge, we propose the resistance switch behavior of the annealed SrTiO_3_-based device is originated from the newly formed conductive nano-networked filaments. This study suggests that the new, simple and easily- operated approach has successfully induced the resistance switch characteristic into the SrTiO_3_ insulator.

## References

[1] Pan F., Gao S., Chen C., Song C., Zeng F. (2014). Recent progress in resistive random access memories: Materials, switching mechanisms, and performance. Mater. Sci. Eng. R Rep..

[2] Cooper D., Baeumer C., Bernier N., Marchewka A., La Torre C., Dunin-Borkowski R.E., Menzel S., Waser R., Dittmann R. (2017). Anomalous Resistance Hysteresis in Oxide ReRAM: Oxygen Evolution and Reincorporation Revealed by In Situ TEM. Adv. Mater..

[3] Wang Y., Shi X., Zhao K., Xie G., Huang S., Zhang L. (2016). Controllable resistive switching in Au/Nb:SrTiO_3_ microscopic Schottky junctions. Appl. Surf. Sci..

[4] Lee K.-J., Wang L.-W., Chiang T.-K., Wang Y.-H. (2015). Effects of Electrodes on the Switching Behavior of Strontium Titanate Nickelate Resistive Random Access Memory. Materials.

[5] Mikheev E., Hoskins B.D., Strukov D.B., Stemmer S. (2014). Resistive switching and its suppression in Pt/Nb:SrTiO_3_ junctions. Nat. Commun..

[6] Rodenbucher C., Menzel S., Wrana D., Gensch T., Korte C., Krok F., Szot K. (2019). Current channeling along extended defects during electroreduction of SrTiO_3_. Sci. Rep..

[7] Schraknepper H., Weirich T.E., De Souza R.A. (2018). The blocking effect of surface dislocations on oxygen tracer diffusion in SrTiO_3_. Phys. Chem. Chem. Phys..

[8] Yang Y., Lu W.D. (2016). Progress in the Characterizations and Understanding of Conducting Filaments in Resistive Switching Devices. IEEE Trans. Nanotechnol..

[9] Marrocchelli D., Sun L., Yildiz B. (2015). Dislocations in SrTiO_3_: Easy to Reduce but Not so Fast for Oxygen Transport. J. Am. Chem. Soc..

[10] Szot K., Speier W., Bihlmayer G., Waser R. (2006). Switching the electrical resistance of individual dislocations in single-crystalline SrTiO_3_. Nat. Mater..

[11] Celano U. (2016). Filamentary-Based Resistive Switching. Metrology and Physical Mechanisms in New Generation Ionic Devices.

[12] Celano U., Gastaldi C., Govoreanu B., Richard O., Bender H., Goux L., Kar G.S., Vandervorst W. (2017). Evidences of areal switching in Vacancy-Modulated Conductive Oxide (VMCO) memory. Microelectron. Eng..

[13] Yang J.J., Inoue I.H., Mikolajick T., Hwang C.S. (2012). Metal oxide memories based on thermochemical and valence change mechanisms. MRS Bull..

[14] Wojtyniak M., Szot K., Wrzalik R., Rodenbucher C., Roth G., Waser R. (2013). Electro-degradation and resistive switching of Fe-doped SrTiO_3_ single crystal. J. Appl. Phys..

[15] Wang Q., Zhang W., Zhang W., Zeng H. (2016). In-situ monitor of insulator to metal transition in SrTiO_3_ by Ar^+^ irradiation. Appl. Surf. Sci..

[16] Psiuk B., Szade J., Szot K. (2016). SrTiO_3_ surface modification upon low energy Ar^+^ bombardment studied by XPS. Vacuum.

[17] Patterson E.A., Major M., Donner W., Durst K., Webber K.G., Rodel J. (2016). Temperature-Dependent Deformation and Dislocation Density in SrTiO_3_ (001) Single Crystals. J. Am. Ceram. Soc..

[18] Pan X.Q., Shuai Y., Wu C.G., Luo W.B., Sun X.Y., Yuan Y., Zhou S.Q., Ou X., Zhang W.L. (2016). Resistive switching behavior in single crystal SrTiO_3_ annealed by laser. Appl. Surf. Sci..

[19] Crespillo M.L., Graham J.T., Agulló-López F., Zhang Y., Weber W.J. (2017). Role of oxygen vacancies on light emission mechanisms in SrTiO_3_ induced by high-energy particles. J. Phys. D Appl. Phys..

[20] Crespillo M.L., Graham J.T., Agulló-López F., Zhang Y., Weber W.J. (2018). Isolated oxygen vacancies in strontium titanate shine red: Optical identification of Ti^3+^ polarons. Appl. Mater. Today.

[21] Crespillo M.L., Graham J.T., Agulló-López F., Zhang Y., Weber W.J. (2017). Correlation between Cr^3+^ Luminescence and Oxygen Vacancy Disorder in Strontium Titanate under MeV Ion Irradiation. J. Phys. Chem. C.

[22] Crespillo M.L., Graham J.T., Agulló-López F., Zhang Y., Weber W.J. (2019). Recent Advances on Carrier and Exciton Self-Trapping in Strontium Titanate: Understanding the Luminescence Emissions. Crystals.

[23] Crespillo M.L., Graham J.T., Agulló-López F., Zhang Y., Weber W.J. (2019). The blue emission at 2.8 eV in strontium titanate: Evidence for a radiative transition of self-trapped excitons from unbound states. Mater. Res. Lett..

[24] Eyvazov A.B., Inoue I.H., Stoliar P., Rozenberg M.J., Panagopoulos C. (2013). Enhanced and continuous electrostatic carrier doping on the SrTiO_3_ surface. Sci. Rep..

[25] Szot K., Speier W., Carius R., Zastrow U., Beyer W. (2002). Localized Metallic Conductivity and Self-Healing during Thermal Reduction of SrTiO_3_. Phys. Rev. Lett..

[26] Cuong D.D., Lee B., Choi K.M., Ahn H.-S., Han S., Lee J. (2007). Oxygen Vacancy Clustering and Electron Localization in Oxygen-Deficient SrTiO_3_: LDA+U Study. Phys. Rev. Lett..

[27] Shablaev S.I., Grachev A.I. (2016). Effect of unsteady-state conduction of a high-resistance SrTiO_3_ crystal containing a network of conductive nanowires. Phys. Solid State.

[28] Liao X.-X., Wang H.-Q., Zheng J.-C. (2013). Tuning the Structural, Electronic, and Magnetic Properties of Strontium Titanate Through Atomic Design: A Comparison Between Oxygen Vacancies and Nitrogen Doping. J. Am. Ceram. Soc..

[29] Gautam S.K., Das A., Ojha S., Shukla D.K., Phase D.M., Singh F. (2016). Electronic structure modification and Fermi level shifting in niobium-doped anatase titanium dioxide thin films: A comparative study of NEXAFS, work function and stiffening of phonons. Phys. Chem. Chem. Phys..

[30] Mi Y.Y., Yu Z., Wang S.J., Gao X.Y., Wee A.T.S., Ong C.K., Huan C.H.A. (2007). Thermal stability of nitrogen-doped SrTiO_3_ films: Electronic and optical properties studies. J. Appl. Phys..

[31] Soriano L., Abbate M., Fernandez A., GonzalezElipe A.R., Sanz J.M. (1997). Chemical analysis of ternary Ti oxides using soft X-ray absorption spectroscopy. Surf. Interface Anal..

[32] Palina N., Annadi A., Asmara T.C., Diao C., Yu X., Breese M.B.H., Venkatesan T., Ariando, Rusydi A. (2016). Electronic defect states at the LaAlO_3_/SrTiO_3_ heterointerface revealed by O K-edge X-ray absorption spectroscopy. Phys. Chem. Chem. Phys..

[33] Bailey T.J., Jha R. (2018). Understanding Synaptic Mechanisms in SrTiO_3_ RRAM Devices. IEEE Trans. Electron Devices.

[34] Yan Z.B., Yau H.M., Li Z.W., Gao X.S., Dai J.Y., Liu J.-M. (2016). Self-electroforming and high-performance complementary memristor based on ferroelectric tunnel junctions. Appl. Phys. Lett..

[35] Mojarad S.A., Goss J.P., Kwa K.S.K., Zhou Z., Al-Hamadany R.A.S., Appleby D.J.R., Ponon N.K., O’Neill A. (2012). Leakage current asymmetry and resistive switching behavior of SrTiO_3_. Appl. Phys. Lett..

[36] Chen X.G., Ma X.B., Yang Y.B., Chen L.P., Xiong G.C., Lian G.J., Yang Y.C., Yang J.B. (2011). Comprehensive study of the resistance switching in SrTiO_3_ and Nb-doped SrTiO_3_. Appl. Phys. Lett..

[37] Adepalli K.K., Yang J., Maier J., Tuller H.L., Yildiz B. (2017). Tunable Oxygen Diffusion and Electronic Conduction in SrTiO_3_ by Dislocation-Induced Space Charge Fields. Adv. Funct. Mater..

[38] Waldow S.P., De Souza R.A. (2016). Computational Study of Oxygen Diffusion along a [100] Dislocations in the Perovskite Oxide SrTiO_3_. ACS Appl. Mater. Interfaces.

[39] Chang Y.-F., Fowler B., Zhou F., Chen Y.-C., Lee J.C. (2016). Study of self-compliance behaviors and internal filament characteristics in intrinsic SiO_x_-based resistive switching memory. Appl. Phys. Lett..

[40] Acharya S.K., Nallagatla R.V., Togibasa O., Lee B.W., Liu C., Jung C.U., Park B.H., Park J.-Y., Cho Y., Kim D.-W. (2016). Epitaxial Brownmillerite Oxide Thin Films for Reliable Switching Memory. ACS Appl. Mater. Interfaces.

